# Temporal asymmetries in auditory coding and perception reflect multi-layered nonlinearities

**DOI:** 10.1038/ncomms12682

**Published:** 2016-09-01

**Authors:** Thomas Deneux, Alexandre Kempf, Aurélie Daret, Emmanuel Ponsot, Brice Bathellier

**Affiliations:** 1 Unité de Neuroscience, Information et Complexité (UNIC), Centre National de la Recherche Scientifique, UPR 3293, F-91198 Gif-sur-Yvette, France; 2 Institut de Recherche et de Coordination Acoustique/Musique (IRCAM), Centre National de la Recherche Scientifique, UMR 9912, F-75004 Paris, France

## Abstract

Sound recognition relies not only on spectral cues, but also on temporal cues, as demonstrated by the profound impact of time reversals on perception of common sounds. To address the coding principles underlying such auditory asymmetries, we recorded a large sample of auditory cortex neurons using two-photon calcium imaging in awake mice, while playing sounds ramping up or down in intensity. We observed clear asymmetries in cortical population responses, including stronger cortical activity for up-ramping sounds, which matches perceptual saliency assessments in mice and previous measures in humans. Analysis of cortical activity patterns revealed that auditory cortex implements a map of spatially clustered neuronal ensembles, detecting specific combinations of spectral and intensity modulation features. Comparing different models, we show that cortical responses result from multi-layered nonlinearities, which, contrary to standard receptive field models of auditory cortex function, build divergent representations of sounds with similar spectral content, but different temporal structure.

## Abstract (web summary)

In humans, sounds that increase in intensity over time (up-ramp) are perceived as louder than down-ramping sounds. Here the authors show that in mice this bias also exists and is reflected in the complex nonlinearities of auditory cortex activity.

## Graphical abstract

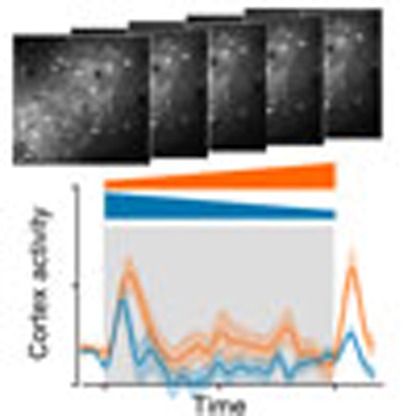

Since the work of von Helmholtz^1^, it is well recognized that sound perception involves frequency decomposition of the acoustic waves by the auditory system. However, the frequency spectrum is not the only characteristic that influences perception and identification of sounds. Psychophysical experiments in audition have shown that temporal features of sounds, that is, the sequence of intensity and frequency variations, are also crucial determinants of perception, not only for sound localization, but also for their identification^2^,^3^,^4^,^5^,^6^. For example, the recognition of sounds from different musical instruments by humans strongly depends on the time-intensity profile of the tones and is strongly impaired by time reversal of the waveform^7^,^8^. Even for simple percepts, such as loudness, temporal features play an important role^9^. Numerous psychophysical experiments have shown that sounds whose intensity is ramping up with time (up-ramps) are globally perceived as louder or changing more in loudness than their time-symmetric opposites (down-ramps). This perceptual asymmetry has been observed for a wide range of sound durations^10^,^11^,^12^,^13^ and is proposed to emphasize approaching sound sources relative to sources moving away^10^ to favour threat detection. The physiological bases of this perceptual asymmetry are yet unknown, but several studies found the activity correlates in later stages of the auditory system. In humans, functional magnetic resonance imaging studies have shown that up-ramps produce stronger BOLD signals than down-ramps already in the auditory cortex^14^,^15^,^16^. Similarly in monkeys, local field potential (LFP) and multi-unit recordings in auditory cortex have demonstrated a positive bias for up-ramps in the global cortical response, consistent with behavioural asymmetries^17^,^18^. Recent single neuron recordings in cat auditory cortex have suggested the existence of a positive bias for up-ramps in non-primates, although this study focused only on very short ramps and found a bias only for the duration of cortical responses^19^.

Although all these results suggest a coding asymmetry between up- and down-ramps, the representation principles of intensity-modulated sounds in auditory cortex and the computational underpinnings of asymmetric responses to sounds are largely unknown, despite their pivotal relevance to the understanding of natural sound perception. Moreover, it is unknown whether the asymmetric perception of intensity-modulated sounds is a shared property of the mammalian auditory system and could be studied with the powerful tools available for a simpler animal model, such as the mouse.

In this report, we combined two-photon calcium-imaging experiments and behavioural assays to show that the positive bias for up-ramping sounds, as compared with down-ramping sounds is present in mice at both the cortical and perceptual level, indicating that this is a general property of the mammalian auditory system. We demonstrate that this bias is the result of profound nonlinearities that go beyond sensory adaptation mechanisms. By analysing the response properties of a large sample of supragranular cortical neurons, we show that the temporal modulations of sounds are encoded by spatially clustered ensembles of neurons that detect specific features about the time course and amplitude of the modulations. Using modelling, we also show that the mechanism underlying the observed perceptual asymmetry is most likely the sequence of nonlinearities implemented in the multi-layered architecture of the auditory system to extract divergent, high-level representations of intensity-modulated sounds.

## Results

### Mean response asymmetry between up- and down-ramps

**Figure 1:**
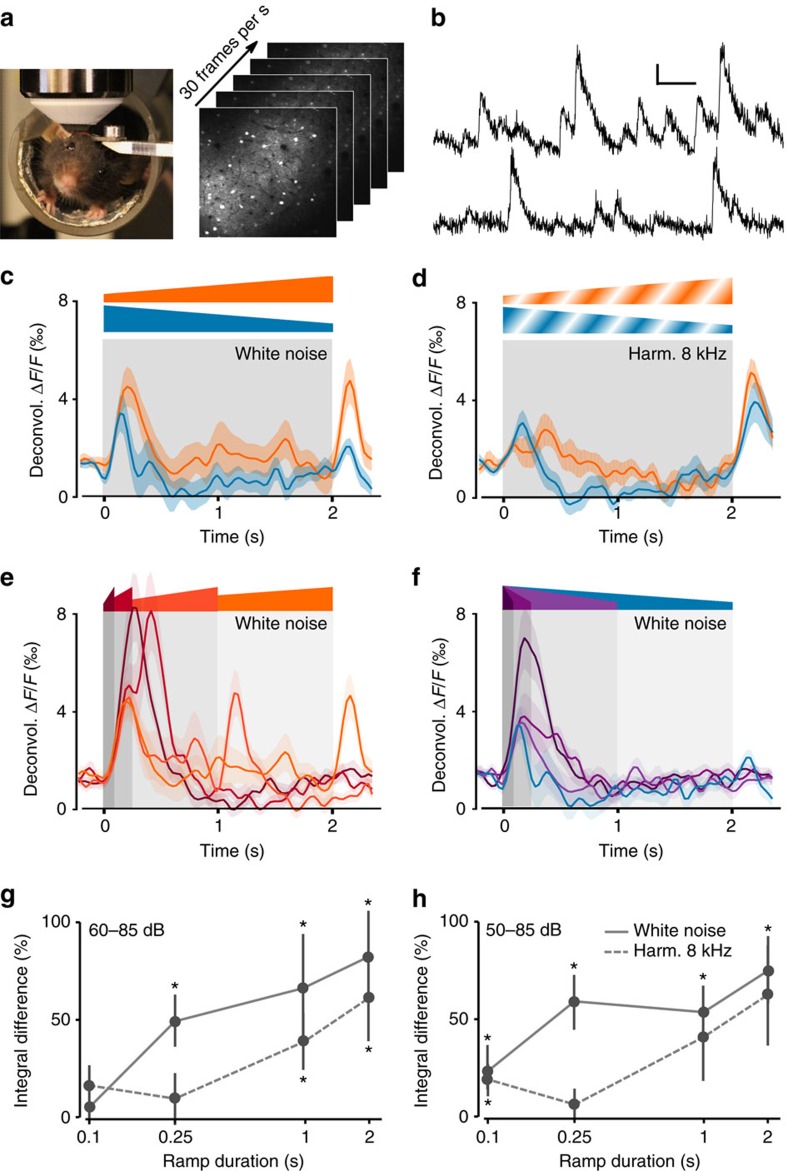
Asymmetry of responses to intensity ramps in mouse auditory cortex. (**a**) Awake head-fixed mouse under the two-photon microscope and an example of a recorded image time series of GCAMP6s labelled neurons in cortical layer 2/3 of the mouse auditory cortex. (**b**) Examples of raw GCAMP6s signals for one neuron (sampling rate: 31.5 Hz). Scale bars, vertical 20% Δ*F*/*F*, horizontal 5 s. (**c**) Mean deconvolved calcium signals (that is, estimated firing rate) for 2 s white noise up- and down-ramps (range 60–85 dB SPL, shading indicates s.e.m. across *n*=15 imaging sessions). (**d**) Same as **c** for 2 s 8 kHz harmonic sound ramps (*n*=13 imaging sessions). (**e**) Responses to white noise up-ramps of 100 ms, 250 ms, 1 and 2 s. (**f**) Same as **e** for down-ramps. (**g**,**h**) Differences of the integrals of response signals between up and down-ramps (for example, integral of the difference of the two mean signals shown in **c**). The differences are normalized by the down-ramp integral. Error bars, s.e.m. When assessed globally (pooling durations together), the integral differences for each intensity range and spectral content was very significantly positive (Wilcoxon signed-rank test, white noise 60–85 dB: *P*=2 × 10^−5^, 50–85 dB: *P*=7 × 10^−9^ *n*=60 measurements; 8 kHz 60–85 dB: *P*=1 × 10^−3^, 50–85 dB: *P*=2 × 10^−3^, *n*=52 measurements). Statistical significance for individual stimuli is assessed across imaging sessions (white noise: *n*=15, 8 kHz: *n*=13) using the single-sided Wilcoxon rank-sum test and a Benjamini–Hochberg correction for multiple testing applied to the 16 tests (*P<0.05).

We first asked if mouse auditory cortex is more strongly driven by up-ramps than by the symmetric down-ramps, although the two signals have equal cumulative physical intensity, as in primate auditory cortex. To answer this question, we performed two-photon calcium imaging in large populations of supragranular neurons of the auditory cortex (imaging depth from 110 μm to 230 μm) expressing the protein calcium sensor GCAMP6s through stereotaxic injection of an AAV-syn-GCAMP6s vector^20^. Mice were awake and held head-fixed under the microscope, using a chronic cranial window and head post implantation (Fig. 1a). This preparation allowed the imaging of multiple fields of view (550 × 540 μm) in the same animal across several days (a different neuronal population was imaged in each session). One or two horizontal locations at one to three vertical positions were sampled in five mice and horizontally remapped (translation and rotation) with respect to each other, using blood vessel patterns. Moreover, a gross identification of auditory cortex subfields^21^ was also obtained based on intrinsic imaging maps, as previously reported^22^ (Supplementary Fig. 1). With this approach, we verified that across 15 imaging sessions in five mice, we densely sampled core subfields of auditory cortex, including A1 (∼40% of the neurons, mice 2, 3 and 4) and the anterior auditory field (anterior auditory field (AAF), ∼45% of the neurons, mice 1, 2 and 5), while we estimate the fraction of neurons from the belt regions (imaged at the ventral or dorsal borders of core fields) to be ∼15% of the neurons. In total, we imaged 4,088 auditory cortex neurons at a rate of 31.5 frames per second using a resonant scanner. Stimuli included a randomized presentation of white noise and 8 kHz harmonic sounds with durations ranging from 100 to 2,000 ms and ramping up or down in intensity. The calcium signals (Fig. 1b) were corrected for neuropil contamination (Supplementary Fig. 2), a step that was essential to ensure the specificity of neuronal response, and temporally deconvolved to more closely track the actual firing rate variations in each identified neuron^22^,^23^ than can be followed with raw calcium signals (Supplementary Fig. 3, but note that deconvolved signals are likely to still contain residual time shifts on the order of tens of milliseconds due to the slow rise time of GCAMP6s).

Averaging the estimated activity of all recorded neurons, we observed that population responses to up-ramps were in many cases larger than for the symmetric down-ramp (Fig. 1c–f). This was particularly evident for the longer white noise ramps (60–85 dB sound pressure level (SPL)) for which the activity was at almost all times larger for the up-ramp than for the down-ramp (Fig. 1c), and this same trend was also clear for the 8 kHz harmonic sound (Fig. 1d). Typical responses to the ramps included onset- and offset-response peaks, which were merged into a single peak for the shortest ramps (Fig. 1c–f; Supplementary Fig. 1). Interestingly, the onset responses were attenuated with increasing ramp duration, which was likely to be due to superposition of onset and offset responses in shorter ramps, but may also be due to some inhibitory process (Fig. 1f). To quantify the asymmetry over the entire time course of the response, we measured the difference of response integrals for up- and down-ramps (Fig. 1g,h). This difference was systematically positive and could be as high as 80±22% (mean±s.e.m., *n*=15 populations) of the average ramp integral for 2 s white noise ramps (60–85 dB; Fig. 1g,h). Individual statistical analysis with correction for multiple testing (Fig. 1g,h), showed significant asymmetry for most ramp parameters, although shorter ramps and 8 kHz harmonic sounds displayed weaker asymmetry that did not reach significance thresholds (Fig. 1g,h). Hence, asymmetries in the global cortical response between up- and down-ramps are clearly present in mouse auditory cortex, as observed in other animal species such as monkeys^17^,^18^. In addition, the direction of the asymmetry is similar to that observed in human sound-level perception assays, although perceptual asymmetry in humans is stronger for harmonic sounds than for white noise^10^,^24^,^25^.

### Linear and adaption models do not explain response asymmetry

These observations raise the question of whether current models of auditory cortex sound encoding can readily explain up- versus down-ramp asymmetries. The responses of auditory cortical neurons are often modelled as linear filters of the sound input, also called spectro-temporal receptive fields (STRFs)^26^,^27^,^28^, which correspond to a two-dimensional (2D) linear filter acting on the sound spectrogram. In this study, we did not characterize the STRFs because we could show mathematically that the response of any STRF filter to our intensity ramps is equivalent to the convolution of the ramp envelope with a linear temporal filter (Supplementary Note 1).

**Figure 2:**
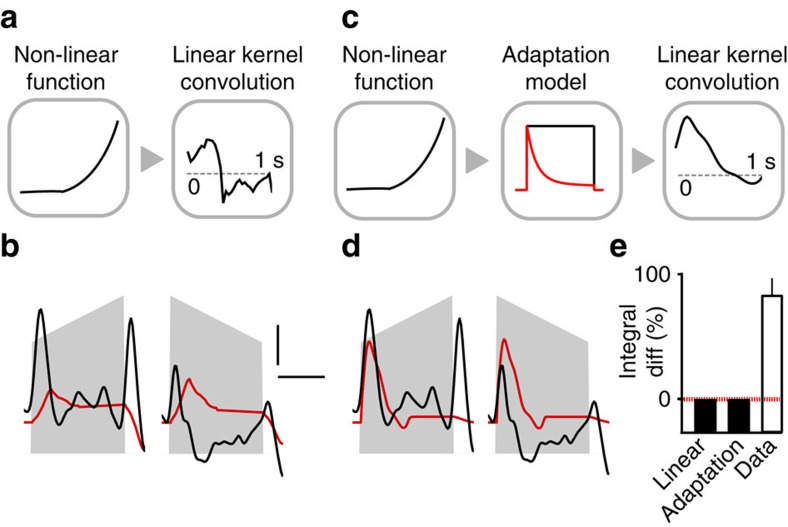
Cortical response asymmetry is a nonlinear effect. (**a**) Sketch of the linear filter model. The input signal is scaled by a nonlinear function (left) and then goes through a linear kernel (right) to obtain the neuronal response. (**b**) Best fit by the linear model of the population responses to the 2 s white noise up- and down-ramps. Scale bars, vertical 0.1% Δ*F*/*F*, horizontal 1 s. (**c**) Sketch of the adaption model. The input signal is scaled by a nonlinear function (left), then undergoes adaptation (middle) and finally passes through a linear kernel (right). (**d**) Best fit by the adaptation model of the population responses to the 2 s white noise up- and down-ramps. (**e**) Integral differences between up- and down-ramps for the linear and adaptation models for any choice of parameters and any ramp waveform (analytical result) versus experimental integral differences for the 2 s white noise ramps.

Moreover, we demonstrated analytically that, as a general rule for any linear filter (or sum of linear filters), the integral of the output is independent of whether the input signal is played forward or backward (Supplementary Note 1). Hence, any linear filter model including STRF models, by construction, predicts equal response integrals for up- and down-ramps. The theorem is also true if a nonlinear scaling function (for example, logarithmic-intensity scaling) is applied to the signal before passing it through the linear filter (Methods; Supplementary Note 1; Fig. 2a). These analytical results can be illustrated by showing the responses to up- and down-ramps of a temporal linear filter optimized to fit the observed cortical data. Despite optimization, the agreement with measured responses was very poor (Fig. 2b). This observation corroborates earlier demonstrations that in general, STRFs fail to explain single neuron responses to a wide range of sounds^29^,^30^,^31^. In addition, here we show that that STRFs are inaccurate for simple intensity modulations, even at the population level.

Auditory cortical responses are known to show strong adaptation^32^ and it was proposed that the STRF model can be combined with a synaptic adaptation model to better fit cortical responses^31^. However, we showed analytically that the adaptation plus STRF model also preserves the equality of input integrals (Supplementary Note 1), so that even the best fit of an adaptation model followed by a linear filter cannot explain the population data (Fig. 2c,d). Therefore, the clear discrepancy between the data and the equality of response integrals predicted by simple or extended STRF models (Fig. 2e) shows that the observed asymmetry between up- and down-ramps is the result of unidentified nonlinearities in the auditory system.

### Asymmetric encoding of up- and down-ramps

**Figure 3:**
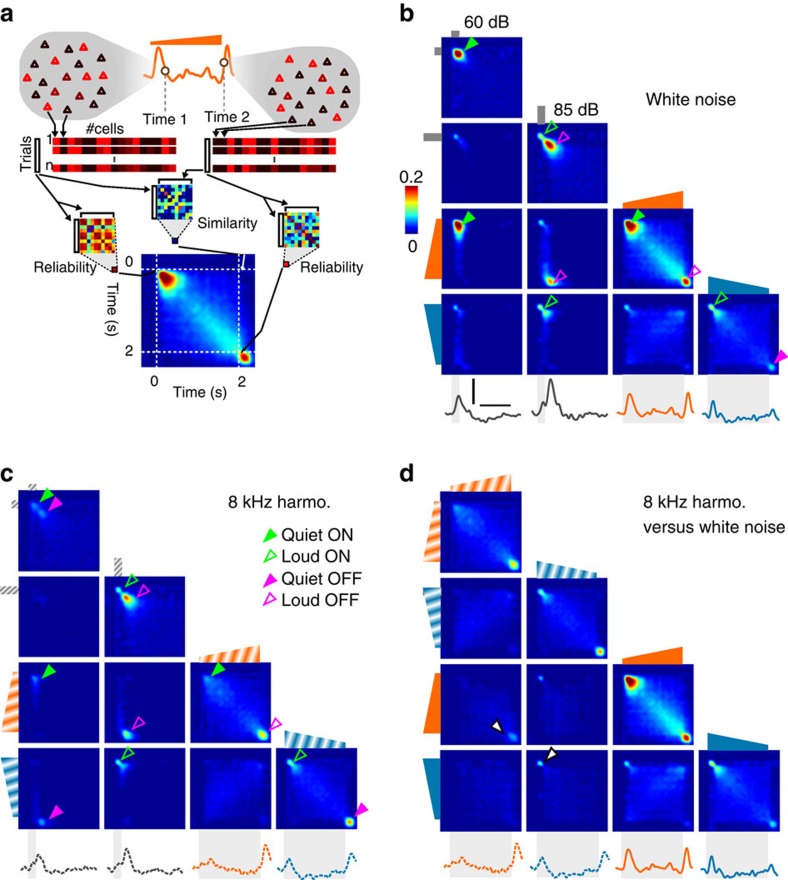
Cortical population dynamics during up- and down-ramps. (**a**) Schematic of the population vector similarity measure (Methods). For this analysis, we pooled all recorded neurons in a pseudo-population. The similarity between two population activity patterns (for example, patterns at time 1 and 2 post stimulus) corresponds to the average of all pair-wise correlations computed across single-trial population vectors. The reliability of a pattern is computed identically based on correlations computed across all single-trial occurrences of this pattern. (**b**) Population similarity matrix across time bins and stimuli for four white noise sounds (60 and 85 dB 0.25 s duration and 60–85 dB 2 s duration up- and don-ramps). Underneath the population firing rate waveforms are shown. The arrowheads on the diagonal indicate distinct activity patterns identified as ‘Quiet ON' (filled green), ‘Loud ON' (empty green), ‘Quiet OFF' (filled magenta) and ‘Loud OFF' (empty magenta). Arrowheads off the diagonal indicate strong similarities between different responses (for example, empty magenta arrowhead indicates similarity between ‘Loud OFF' activity patterns observed after the 85 dB 250 ms sound and after the 60–85 dB up-ramp). Scale bars, vertical 0.4% Δ*F*/*F*, horizontal 1 s. (**c**) Same as **b** for the harmonic 8 kHz tone. (**d**) Same as **b** and **c**, but the response to the white noise and 8 kHz ramps are compared.

To better understand the origin of the observed coding asymmetry, we analysed the sequence of global cortical population activity patterns produced during up- and down-ramp presentations. To do so, we defined a measure of similarity between population activity patterns elicited at different time points of the stimulus presentation (Fig. 3a; Methods). To evaluate the similarity of population responses during the up- and down-ramps, we plotted similarity matrices for all time points of the sound presentations, including similarity across both ramps (Fig. 3b,c). This allowed us to identify four different types of population patterns for each sound quality tested (white noise, Fig. 3b; 8 kHz, Fig. 3c). These included a response typical of the up-ramp onset, which was identical to the onset response to a 250 ms constant intensity sound played at the ramp start level (filled green arrowheads). We termed this response type as ‘quiet ON'. A reproducible response pattern was also seen at the up-ramp offset, which was almost identical to the offset response to a 250 ms constant intensity sound played at the ramp end level. We termed this response type as ‘loud OFF'. For the down-ramp, we observed a ‘loud ON' response pattern immediately after onset, and a ‘quiet OFF' response pattern immediately after offset, which was more clearly evident for the 8 kHz sounds. Although some residual similarity was observed between the ‘loud ON' and ‘quiet ON' response types, these patterns corresponded to a specific encoding of multiple sound features, including not only the direction (ON versus OFF), but also the intensity (quiet versus loud) of fast variations. Besides these salient responses to transients, specific but more variable activity patterns were produced during the slow ramping phase of the stimuli, most visibly for the white noise up-ramp (Fig. 3b). This complex time-intensity code leads to a very asymmetric response sequences for longer up- and down-ramps, as seen in the matrices comparing up- and down-ramp responses (Fig. 3b,c). Importantly, along with time-intensity coding, we also observed (as expected) sound quality coding, as the four ON and OFF response patterns for white noise were distinct from the ON and OFF response patterns for 8 kHz sounds, despite some similarities for loud ON and OFF patterns (Fig. 3d). Altogether, this analysis showed that the population encoding of multiple sound parameters (in particular the level and direction of intensity modulations) leads to an asymmetric cortical representation of up-ramping and down-ramping sounds.

### Multifeature coding of intensity modulations

**Figure 4:**
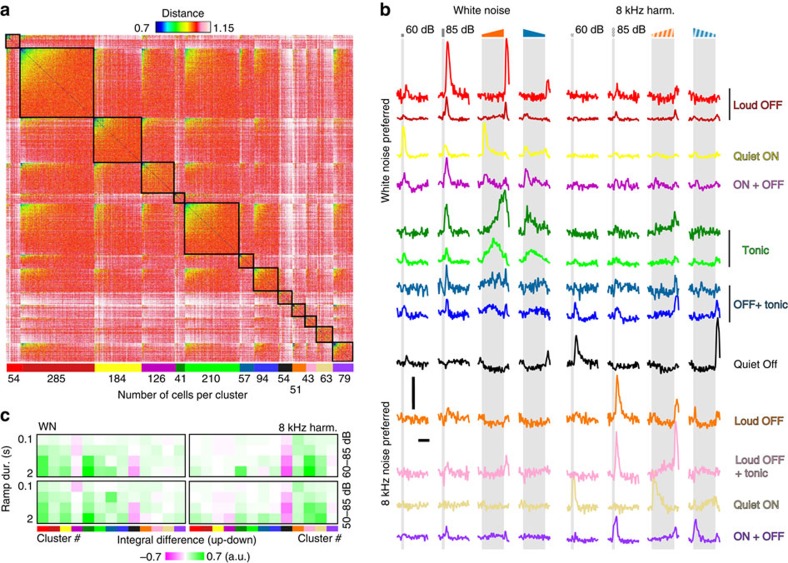
Clustering of single neuron population responses. (**a**) Distance matrix for the 1,341 clustered neurons. The metric used is *d*=1−cc, where cc stands for the Pearson correlation coefficient between response traces. The identified clusters are delineated by a black square and labelled at the bottom of the matrix by a coloured bar under which the number of cells in the cluster is indicated. Within each cluster the cells are sorted according to their mean distance with all other cells of the matrix. The gradient of distances within each cluster reflects the heterogeneity of the signal-to-noise ratio across cells. More reliable cells are on the left, less reliable cells on the right. (**b**) Mean response profiles of the 12 identified clusters to four white noise and 8 kHz harmonic sounds (60 and 85 dB 0.25 s duration and 60–85 dB 2 s duration up- and down-ramps). Scale bars, vertical 4% Δ*F*/*F*, horizontal 1 s. (**c**) Average absolute integral differences between up- and down-ramps for each cluster, ramp intensity ranges and durations.

To understand the functional properties of single neurons underlying the observed population code, we first aimed to determine the main types of individual responses present in the data set and their distributions. We performed a hierarchical clustering of significant single neuron responses, using the similarity of temporal response profiles across neurons as a metric (Methods). First, ∼63% of the neurons had response profiles non-discriminable from noise and were classified as weakly or non-responsive consistent with the reported sparseness of auditory cortex response in awake rodents^33^. For the remaining 1,341 neurons, we obtained 13 clusters displaying different average response profiles (Fig. 4a,b). However here, we used clustering mainly to organize the data set, and not to identify fully distinct clusters: although some clusters were clearly separated from each other, others represented variations of one another in a continuum. Clusters were first distinguished by their selectivity to sound quality. Although several clusters responded both to white noise and 8 kHz sounds, seven of them showed preference (stronger responses) for white noise and six for 8 kHz sounds. Another important difference between clusters was their sensitivity to particular intensity modulation features. Eight clusters (70% of the clustered population) seemed to respond to a single precise feature of sounds. These included ‘loud' offsets as characterized by ‘OFF' responses to up-ramps, but not down-ramps and to loud, but not quiet constant sounds as observed in three clusters (Fig. 4b, Loud OFF, 30% of the cells). We also observed two clusters of cells responding to ‘quiet' onsets as characterized by ‘ON' responses to up-ramps but not down-ramps and to quiet, but not loud constant sounds (Fig. 4b, Quiet ON, 18% of the cells) and a small population was found responding to ‘quiet' offsets (Fig. 4b, Quiet OFF, 4% of the cells). In addition, 18% of all clustered neurons (two clusters) responded in a tonic manner to the loud (or intermediate loud) part of long ramps (Tonic). In contrast to these very specific clusters, we found five clusters signalling several intensity modulation features, including two clusters responding both to on- and offsets, (Fig. 4b, ON+OFF, 15% the cells) and three clusters responding both to loud offsets and the central loud part of the ramp (Fig. 4b, Loud OFF+Tonic, 15% of the cells). All these cluster subtypes were divided into one or two white noise or 8 kHz preferring cluster, except for Quiet OFF (8 kHz only) and Tonic (white noise only). Strikingly however, we did not find Loud ON clusters despite the presence of a specific Loud ON pattern at the population level (Fig. 3). In fact, Loud ON patterns correspond to the response of ON+OFF neurons alone. Therefore, the four identified on- and offset population patterns, as well as the population pattern observed during slow up-ramping (Fig. 3) all reflected the combined activation of several neuronal types detecting different features of the intensity-modulated sounds. Interestingly, loud and quiet offsets, as well as quiet onset patterns contained cells very specific to the associated feature, while loud onset patterns were reflected by the activity of the less specific ON+OFF neurons. Importantly, almost all of these neuronal types showed asymmetric responses to up- versus down-ramps, but only three clusters (239 out of 1,341 neurons, quiet OFF and ON+OFF) preferred down-ramps for their preferred spectral signal (white noise or 8 kHz, Fig. 4c). This sparser encoding of time-intensity features specific to down-ramps explains the response asymmetry at the population level.

### Spatial organization of feature specific neurons

**Figure 5:**
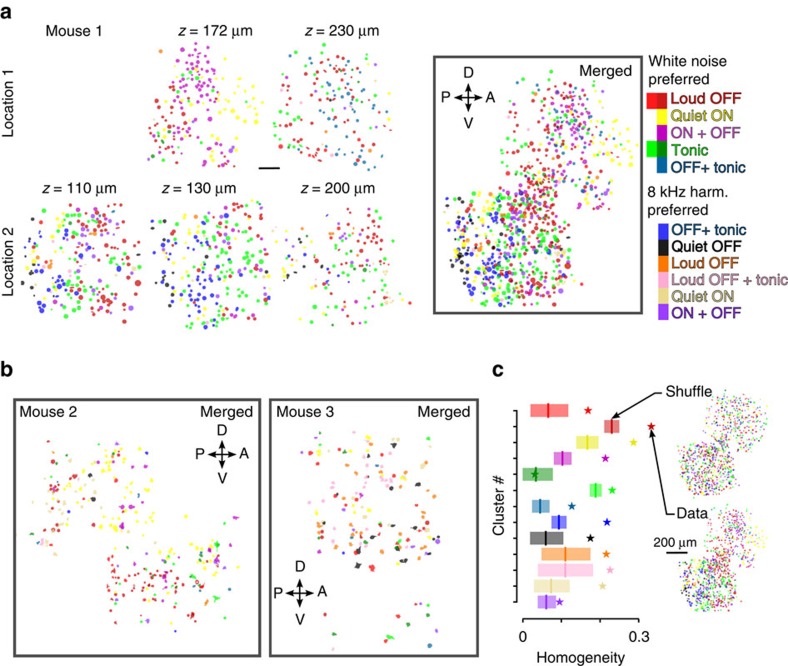
Functional cell types correspond to cell assemblies clustered in space. (**a**) Localizations of the cells belonging to the different identified clusters, colour-coded as in Fig. 4 (see colour bar on the right), in five imaging sessions performed at two different horizontal localizations and different depths (*z*) across several days in mouse 1. On the right, the relative localization of all cells is shown in a horizontally mapped *z* projection. Scale bar, 100 μm. (**b**) Horizontally mapped *z* projection for mouse 2 and mouse 3 (four imaging sessions each, Supplementary Fig. 4). (**c**) Each star represents the value of a homogeneity index calculated across three mice for each of the 13 clusters (same colour code as in **a**). The vertical lines represent the value expected if each cluster was homogenously spread in space obtained by shuffling and the shaded area is the 95% confidence interval. Maps obtained before and after shuffling are shown for one example mouse: note that cell shufflings are operated within individual mice, but homogeneity indexes averaged over neurons across all mice.

Next, we investigated the spatial organization of the clusters, by colour coding them in the imaging fields of view. We observed a relatively spread spatial distribution of the different cell types across imaging fields and mice (Fig. 5a,b). However, in some imaging fields it was also clear that, despite some spatial intermingling, the clusters were unevenly distributed (for example, mouse 1 Fig. 5a, Supplementary Fig. 4). In three out of five mice, we obtained multiple imaging sessions across different days in contiguous regions, either situated in nearby horizontal positions or at different cortical depths. In mouse 1 (Fig. 5a) and 2 (Fig. 5b) but less in the more sparsely responding region recorded in mouse 3 (Fig. 5b), we also observed that the regions richer for one cluster were consistent across recording depths (for example, 8 kHz OFF+Tonic or Quiet OFF, Fig. 5a) and were, in some cases, horizontally continuous (white noise Loud OFF and Tonic clusters, Fig. 5a; Quiet ON, Supplementary Fig. 4a). To quantify spatial clustering, we computed for each cluster a homogeneity index representing the average fraction of neighbouring cells within a 30 μm radius that belonged to the same identified cluster (radius size effect are shown in Supplementary Fig. 4) and compared it with maps, in which cluster identity was randomly shuffled within each mouse (for example, inset Fig. 5c). For 12 out of 13 clusters, homogeneity was significantly higher than in shuffled maps (*P*<0.05, Benjamini–Hochberg correction for multiple testing, *n*=3 mice; Fig. 5c). Although we did not systematically investigate this point, comparison between neurons putatively located in AAF or A1 in the same animal (for example, mouse 2) did not reveal obvious differences in stimulus responsiveness and showed a similar distribution of cluster type was observed in both subfields. Spatial clustering was very significant in each individual mouse (Supplementary Fig. 4).Together, these analyses show that the coding of time-intensity features is spatially organized in the mouse auditory cortex.

### Data-driven model of sound encoding nonlinearities

**Figure 6:**
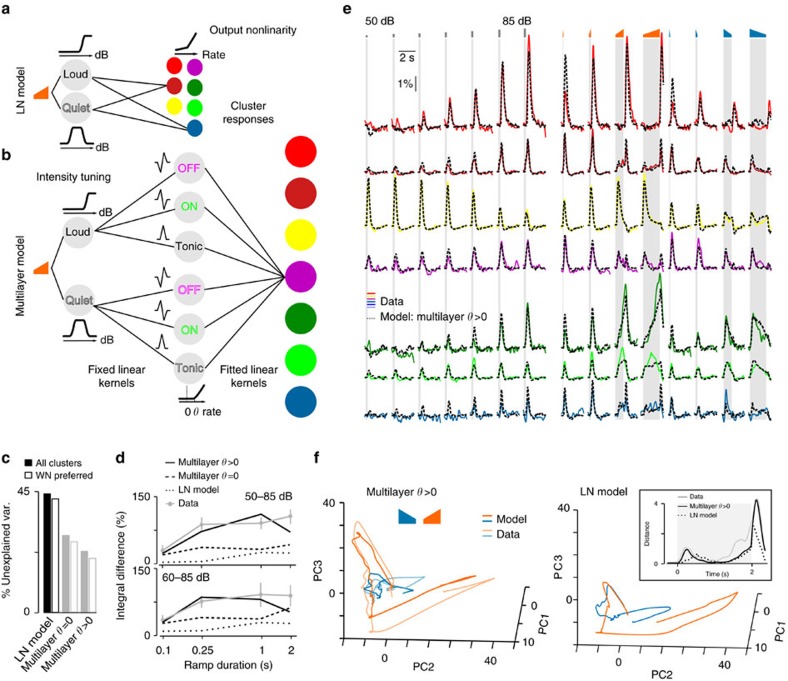
Phenomenological model of intensity modulation coding. (**a**) Linear–nonlinear (LN) model (linear filters+output nonlinearity) applied to two different ‘intensity channels' obtained by nonlinear scaling of the input for intensity tuning. (**b**) The multilayer model with: (1) intensity channel as in **a**, (2) six fixed linear filters with a rectifying nonlinearity (threshold=*θ*) and (3) linear sum of the feature detector outputs filtered by fitted kernels. (**c**) Fraction of unexplained variance for all clusters (filled bars) or for the seven clusters preferring white noise (empty bars) after fitting the LN model, and the full multilayer model with a fixed (multilayer *θ*=0) or a fitted (multilayer *θ*>0) rectifying threshold. (**d**) Normalized difference of the up- and down ramp responses for the clustered data (1,341 neurons, *n*=13 imaging session) and the different fitted models as in **b**. (**e**) Fit of the multilayer model (*θ*>0) to the responses of the six identified clusters that show preferred response to white noise (note that all 12 clusters where fitted by the model). Sounds are white noise: 250 ms constant (seven intensities) and 60–85 dB up- and down-ramps. Scale bars, vertical 1% Δ*F*/*F*, horizontal 2 s. (**f**) Trajectories of the population responses to the 2 s white noise up- (orange) and down-ramps (blue) obtained for the fitted LN (right), the multilayer model and the data (left). The 13-dimensional data and model outputs are plotted in the space of the three first-principle components of the data. The trajectories are more divergent for the multilayer model than for the LN model, as corroborated by distance between the two trajectories at every time point (inset).

To better understand what types of nonlinearity could be responsible for the asymmetric encoding of up- and down-ramps, we searched for models that could account for our observations, including asymmetric response integrals, on- and offset responses and specific sound intensity coding in certain neurons. The simpler nonlinear models applied to both auditory^34^,^35^ and visual^36^,^37^,^38^ systems combine a linear filter (receptive field) with a nonlinear function that is expected to capture output nonlinearities, such as the spike threshold. Initially, we tried to fit such a linear–nonlinear model (LN model; Fig. 6a) to all white noise responses of the 13 neuronal clusters. Because the data showed clear intensity tuning split into groups of cells that either respond to low or high-amplitude changes, we first assumed that the sound envelope is encoded through a ‘quiet' and a ‘loud' channel modelled with two different nonlinear scaling functions applied to the input intensity (Fig. 6a), whose parameters were optimized for each tested model. The modelled response of each cluster was thus the sum of two linear filters applied to each of these channels followed by a nonlinearity (Fig. 6a; LN model; Methods). Using this approach, the best fit on our training stimulus set (Methods) was unable to explain 44.2% of the total variance of the responses to the test stimulus set (Fig. 6c). More importantly, the LN model was unable to reproduce the magnitude of the asymmetry between up- and down-ramp responses (Fig. 6d). We thus concluded that the structure of LN-type models does not reflect the computations underlying the observed cortical responses. Importantly, this implies that neither intensity tuning nor output nonlinearities are sufficient to explain the observed cortical asymmetries.

The main reason for the failure of LN-type models is their inability to account for encoding the combination of certain features, as observed in a large number of recorded neurons. LN-type models, for example, fail to explain the responsiveness of visual cortex complex cells to ON and OFF oriented edges, a property better modelled by the summation of at least two inputs (ON+OFF)^38^,^39^. On the basis of this idea, we added to our LN model a ‘middle layer' of units computing simple linear features and followed by a rectifying nonlinear function (‘multilayer' model; Fig. 6b). The features included tonic responses (transmission of their input) or first-order derivatives (either positive for ON-units or negative for OFF-units). In this multilayer model, cortical responses were then modelled as the sum of fitted linear kernels applied to these six nonlinear units (Fig. 6b; see Methods and Supplementary Fig. 5 for the fitted kernels). In comparison with the LN model, this architecture failed to explain 28.8% of the response variance in the test set, when the threshold of the rectifying nonlinearity was set to zero, and failed to explain only 23.1% when it was fitted to an optimal positive value (Fig. 6c; *θ*>0), without any further output nonlinearity. When quantifying only from the six clusters preferring white noise, which have a larger signal-to-noise ratio (SNR), the unexplained variance even dropped to 20.4% (Fig. 6c; *θ*>0) with a visually striking fit to the data (Fig. 6e). The multilayer model also closely reproduced the asymmetry of population integrals (Fig. 6d). Moreover, the kernels obtained with the multilayer model were smooth positive or negative transient functions with decay time constants of 100–200 ms and thus compatible with long, polysynaptic, post-synaptic potentials (Supplementary Fig. 5). This supports the functional plausibility of the chosen architecture despite its oversimplification with respect to the underlying biological network.

Together, this analysis shows that more than one nonlinear processing layer is required to explain the multifeature code observed in auditory cortical neurons. Interestingly, the result of this complex transformation of the auditory inputs is an asymmetric, divergent representation of temporally symmetric sounds, as can be seen when comparing the population trajectories for the data and the multilayer model with trajectory results from the one-layer LN model (Fig. 6f). Interestingly, this computational scheme and the overrepresentation of particular features also allows differential boosting of the overall saliency of up-ramps based on their temporal profile.

### Up-ramps are behaviourally more salient than down-ramps

**Figure 7:**
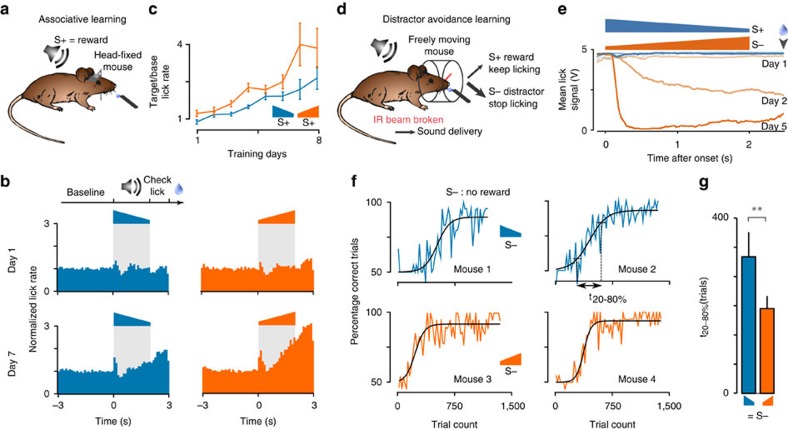
Up-ramps are behaviourally more salient than down-ramps. (**a**) Sketch of the head-fixed sound-reward association task. (**b**) Histograms of lick rates normalized to the baseline rate during the first and seventh days of training to the up- (right) and down-ramps (left). Average across all mice (*n*=6 per group). (**c**) Ratio of the post- and pre-stimulus lick rates over training days for the up- (blue) and down-ramps (orange) (mean±s.e.m.), showing increased sound-locked licking for up-ramps, Friedman test, *P*=3.7 × 10^−10^, *n*=6 per group). (**d**) Schematic of the distractor avoidance learning task. Freely moving mice first learn to lick at a spout after an S+ sound to get a reward, then an S− sound is added and mice learn to stop licking after this sound. (**e**) Typical average infrared beam break signal (5 V=beam broken, 0 V=beam intact) with respect to S+ and S− sound onsets for a mouse on the first, second and fifth training days. (**f**) Examples of global performance learning curves (mean of S+ and S− performance) for the Go/NoGo distractor avoidance task. (**g**) Learning phase duration when either the down- (left) or the up-ramp (right) is the S− stimulus (mean±s.e.m., *n*=12 per group, *P*=0.0046, Kolmogorov–Smirnov test). The learning phase duration if defined as the time necessary to go from 20 to 80% of maximum performance above chance level (that is, >50% correct) and is measured on the sigmoid fitted to the learning curve.

Are the nonlinearities observed in cortical sound encoding actually reflected in the perceived saliency of up- and down-ramps in mice? To answer this question, we first used the general observation that more salient stimuli lead to a faster associative conditioning^40^,^41^. We trained two groups of water-deprived, head-fixed mice to lick after ramping sounds to get a water reward (Fig. 7a). Although on the first training day, both groups of mice licked almost irrespective of the sound cue, after seven training days, lick probability increased during and after sound presentation, following a similar profile when either up- or down-ramps were used as a cue (Fig. 7b). However, the ratio between lick rate after sound offset and before sound onset increased faster for the group cued with a 2 s white noise up-ramp (60–85 dB SPL) than for the group cued with the symmetric down-ramp (Fig. 7c). This suggests that, in the context of this task, the up-ramp is more salient than the down-ramp, which results in faster learning. Given the duration of the ramps used in this task, it is unclear whether the up-ramp is more salient overall, or if it is only its terminal high-intensity part that most closely signals the reward (mice had to do at least one lick following sound offset to get a reward).

To test whether the earlier part of the up-ramp is also more salient, as suggested by cortical imaging, we used another associative task. In this task, freely moving mice first rapidly learned to lick at a tube after an S+ ramp to get a reward. Then, after 4 days, a non-rewarded S− ramp was introduced and mice learned to avoid licking following this new sound (Fig. 7d). We observed that, in this task, the response to the S− ramp spontaneously occurred close to sound onset (Fig. 7e). Moreover, because the S+ is already associated with licking, the association between S− and lick-avoidance is rate-limiting for the overall discrimination. Consequently, a learning speed analysis in this task makes it possible to assess the respective perceptual saliency of ramp onsets when comparing groups avoiding the up- or the down-ramp. In accordance with the cortical population response, the rise of individual learning curves was significantly shorter (192±28 trials, *n*=12 mice), when the 2 s 60–85 dB white noise up-ramp was the S− compared with the opposite situation (310±56 trials, *n*=12 mice, *P*=0.0046, Kolmogorov–Smirnov test; Fig. 7g). Note that the individual learning curves for this task typically display a delay period during which performance stays at 50% (licking occurs both on S+ and S− sounds) followed by a learning phase, in which mice start to avoid the S− sound^42^ (Fig. 7f), as observed in many learned behaviours^43^. Hence, we measured rise duration on each individual curve from the end of the delay period to the end of the learning phase.

Taken together, this data indicates that the 2 s up-ramp is behaviourally more salient than the down-ramp both in its initial and terminal phase as predicted by our cortical activity measurements (Fig. 1c). Hence, the asymmetric encoding of up- and down-ramps is reflected in the perceptual choices of the mouse.

## Discussion

In this study, we demonstrate three important points. First, we show that to explain the asymmetric encoding of up- versus down-ramping sounds in auditory cortex, nonlinear processes are required (Figs 1 and 2). Several studies have already shown that auditory cortex is nonlinear through the limitations of linear models such as STRFs in approximating the responses of single neurons in the auditory cortex^29^,^30^. However, neither the type nor the magnitude of the nonlinearities nor their consequences for perception are well characterized. With respect to magnitude and perceptual impact, our study provides a clear example that nonlinearities of the auditory system are so large that they produce population-scale differences in the cortical responses that correlate very well with the asymmetric perceptual saliency of up- and down-ramps observed both in mice (Fig. 7) and humans^10^. With respect to the type of nonlinearities implemented by the auditory system, among the variety of models proposed in the past^31^,^34^,^35^,^39^, our study provides an important constraint. We show that simple nonlinear mechanisms operating at the output of a single layer, such as adaptation or nonlinear output functions (LN models) can account neither for the combinations of different nonlinear features observed in the responses of single neurons, nor for the global asymmetry of cortical responses to up- and down-ramps.

Several studies have already shown the limits of LN models, and have successfully proposed extensions, including nonlinear input scaling or frequency coding functions^44^, adaptation^31^ or gain control mechanisms^45^. Here we show that despite their importance, some of these nonlinearities (nonlinear input scaling, adaptation) are alone insufficient to explain up- versus down-ramp asymmetries (Fig. 2). Instead, we propose that a sequence of nonlinearities embedded in multiple processing layers, as we exemplify in Fig. 6, is required to explain asymmetric coding of intensity-modulated sounds. This conclusion is further supported by the higher fitting accuracy, for various sets of sounds, obtained with other models simulating two layers^35^ or expanding linear models to second order terms^34^. Indeed, these models try in essence to locally approximate complex multilayer architectures, potentially even more complex than the one we propose to account for our specific stimulus set.

Although our conclusions constrain the generic type of computations underlying auditory processing, the biological implementations of such computations could be very diverse. Multilayer architectures computations could be, for example, implemented by a sequence of nonlinearities in the ascending auditory pathway through which increasingly complex features are produced. The observation of off-responses in the auditory thalamus both in mice^46^ and guinea pigs^47^ is in support of this view. As these neurons could be a major contributor to cortical offset responses, it would be interesting to investigate if the dichotomy between quiet and loud offsets (as we observed for 8 kHz sounds in cortex) is also already present in the thalamus. However, the multilayer structure of our model could in principle also reflect cellular nonlinearities (for example, nonlinear dendritic integration can implement two processing layers^48^) or even recurrent connections within or across the networks of cells, corresponding to the functional clusters we identified^49^,^50^.

The second point demonstrated in our study is that even stimuli as simple as up- and down-ramping sounds can reveal novel encoded features in the auditory system, and challenge current models. In particular, we established that sound intensity modulations are encoded in the auditory cortex by multiple neuronal populations that respond to unexpectedly complex combinations of sound features that not only include frequency, but also direction of modulation (for example, onset and offset) and sound level (Fig. 4). A large fraction of cells code very precise feature combinations (for example, offset of a loud white noise sound, or onset of a more quiet sound; Fig. 4), whereas larger groups of cells encode multiple feature combinations (for example, offset and steady part of a loud sound; Fig. 4). Previous reports have described tuning to sound intensity^35^,^51^ or to specific temporal features, such as on- and off-responses^52^ or the rate of click trains^53^, but these were often described independently of other features^51^ or under particular anaesthesia^52^, and so could not be integrated in a general coding scheme. Here we show that these features are combined with each other in the same neurons of the auditory cortex, potentially encoding higher-level perceptual tokens. We also show that some specific feature combinations are favoured, in particular the ‘quiet' onset and ‘loud' offset present in up-ramps (Quiet ON, Loud OFF and Loud OFF+Tonic; Fig. 4). This explains the observed asymmetry. It is noteworthy that, both in the data and the multilayer model, 2,000 ms ramps globally generate larger asymmetry than 100 ms ramps (Figs 1 and 6d) although the same on- and offsets are present for both durations. The reason for this discrepancy can be hypothesized based on the multilayer model simulations. In the multilayer model, the units detecting elementary on- and offsets (middle layer, Fig. 6b) are thresholded (parameter *θ*). In longer ramps, because the central slope is shallow, its contribution to the activity is subthreshold when *θ*>0. In shorter ramps, the central slope is much steeper and this impacts the activity of on- and offset units that partially compensates for the asymmetry (note that when *θ*=0, there is no asymmetry discrepancy between shorter and longer ramps, Fig. 6d).

As a striking side result of our study, we also show that neurons encoding the same feature combination are non-randomly organized across the supragranular layer of the auditory cortex and tend to cluster spatially (Fig. 5; Supplementary Fig. 4), forming a complex multifeature map. However, the structure of this map seems more sparse and diffuse than expected for a purely columnar architecture. This observation is consistent with previous reports inferring^54^ or describing^22^ iso-functional intermingled subnetworks in mouse auditory cortex, as well as with reports indicating that strongly responsive, information-rich cells are sparse in auditory^33^ and other cortical areas^55^,^56^. Given the large number of feature combinations that could possibly exist, but that we did not test in this study, further investigation will be required to reveal the exact organization of these subnetworks and their relationship to tonotopic organization. Nevertheless, our gross localization of imaging fields using intrinsic imaging indicates that these subnetworks exist within tonotopic fields (Supplementary Figs 1 and 4).

The third conclusion of our study is that the multifeature code demonstrated in mouse auditory cortex, and most likely resulting from the multilayer nonlinear architecture of the auditory system, allows the encoding of two distinct temporal modulations of the stimulus with divergent activity patterns, unlike models with a single-output nonlinearity. This fact is evident when comparing the population activity trajectories produced by the fitted LN and nonlinear feature models, respectively (Fig. 6f). It suggests that the purpose of the nonlinearities implemented throughout the auditory system is to produce easily separable representations of distinct temporal sound intensity modulations. Remarkably, neural networks using multiple layers endowed with linearly filtered inputs and a rectifying nonlinearity are capable of generating complex representational features permitting impressive speech or object recognition performance^57^. It seems that such networks implement symmetry-breaking principles that are important for perception. Our results provide novel evidence suggesting that these principles are also at the source of the strikingly distinctive percepts generated by sounds bearing identical spectral content, but different temporal dynamics^2^,^3^.

## Methods

### Animals

All mice used for imaging and behaviour were 6–16-week-old male C57Bl6J mice. All animal procedures were approved by the French Ethical Committee (authorization 00275.01).

### Two-photon calcium imaging in awake mice

At least 3 weeks before imaging, mice were anaesthetized under ketamine medetomidine. The right masseter was removed and a large craniotomy (5 mm diameter) was performed above the auditory cortex. We then performed three injections of 150 nl (30 nl min^−1^), AAV1.Syn.GCaMP6s.WPRE virus obtained from Vector Core (Philadelphia, PA, USA) and diluted × 10. The craniotomy was sealed with a glass window and a metal post was implanted using cyanolite glue followed by dental cement. Two days before imaging, mice were trained to stand still, head-fixed under the microscope for 10–20 min per day receiving small sucrose rewards. Then mice were imaged 1–2 h per day. Imaging was performed using a two-photon microscope (Femtonics, Budapest, Hungary) equipped with an 8 kHz resonant scanner combined with a pulsed laser (MaiTai-DS, SpectraPhysics, Santa Clara, CA, USA) tuned at 920 nm. Images were acquired at 31.5 Hz during blocks of 42 s during that randomly chosen sounds were presented with 2.5 s intervals. Blocks were interleaved by an 18 s pause repeated until all sounds were played 20 times. White noise and harmonic (8 kHz+the five first odd harmonics with a 1/(2*n*+1)^2^ spectrum) sounds were played in 23 different intensity modulations, including seven constant sounds of 250 ms at (50, 55, 60, 65, 70, 80 and 85 dB SPL), eight up- and down-ramps between 50 and 85 dB SPL with four durations (0.1, 0.25, 1 and 2 s) and eight up- and down-ramps between 60 and 85 dB SPL. In two imaging sessions (431 neurons), only white noise sounds were tested. All sounds were delivered at 192 kHz with a NI-PCI-6221 card (National Instrument) driven by Elphy (G. Sadoc, UNIC, France) through an amplifier and high-frequency loudspeakers (SA1 and MF1-S, Tucker-Davis Technologies, Alachua, FL). Sounds were calibrated in intensity at the location of the mouse ear using a probe microphone (Bruel & Kjaer).

### Intrinsic optical imaging recordings

To localize the calcium-imaging recordings with respect to the global functional organization of auditory cortex, we performed intrinsic optical imaging experiments under isoflurane anaesthesia (1%). The brain and blood vessels were illuminated through the cranial window by a red (intrinsic signal: wavelength=780 nm) or a green (blood vessel pattern: wavelength=525 nm) light-emitting diode. Reflected light was collected at 20 Hz by a charge-coupled device camera attached to a macroscope. The focal plane was placed 400 μm below superficial blood vessels. A custom-made Matlab program controlled image acquisition and sound delivery. We acquired a baseline and a response image (164 × 123 pixels, ∼3.7 × 2.8 mm, image shown in Supplementary Fig. 1 are cropped around the sound responsive area) corresponding to the average images recorded 3 s before and 3 s after sound onset, respectively. The change in light reflectance (Δ*R*/*R*) was computed then averaged over the 20 trials for each sound frequency. A 2D Gaussian filter (*σ*=45.6 μm) was used to build the response map (Supplementary Fig. 1). Sounds were trains of 20 white noise bursts or pure tone pips (4,8,16 and 32 kHz) separated by 20 ms smooth gaps.

### Data analysis

Data analysis and modelling was performed with custom-made Matlab and Python scripts available upon request, as well as the data sets. Only recordings performed within auditory cortex as assessed with intrinsic imaging were included in the imaging. Sample size (∼4,000 neurons) was chosen to obtain a representative sampling of auditory cortex as assessed in a previous study^22^. All images acquired during a session were registered by horizontal translation to a template image to correct for motion artefacts (all sessions with visible *z* motion were discarded). Regions of interest were then manually selected on the whole cell bodies of visually identifiable neurons and the mean fluorescence signal *F*(*t*) was extracted for each region. We also estimated the local neuropil signal *F*_np_(*t*) for each neuron. Briefly, (Supplementary Fig. 2a,b) we computed ‘filled-in' neuropil signal frames Y(*t*) by spatially smoothing every data frame X(*t*) with a Gaussian spatial kernel g (*σ*=170 μm) after excluding the neuron's region-of-interests represented by a masked binary image M. This is done using the formula: Y(*t*)=(X(*t*).M)⊗g/(M⊗g), in which ‘.' and ‘/' denote the element-wise multiplication and division and ⊗ is the spatial 2D convolution. Then for each neuron, we computed the neuropil corrected fluorescence signal *F*_c_(*t*)*=F*(*t*)−*0.7 F*_np_(*t*), where *F*_np_(*t*) is the mean value of Y(*t*) in the neurons' region of interest. The 0.7 correction factor was chosen according to the calibration made in another study^20^ for GCAMP6s in mouse visual cortex, but we could visually verify that for our data neuropil contamination was removed with very little artefact while neuron-specific responses were preserved (Supplementary Fig. 2c).

Baseline fluorescence *F*_0_ was calculated as the minimum of a Gaussian-filtered trace over the 42 s imaging blocks and fluorescence variations were computed as *f*(*t*)*=*(*F*_c_(*t*)−*F*_c0_*)/F*_c0_. The approximate time course of the firing rate was estimated using temporal deconvolution as *r*(*t*)*=f*′(*t*)*+f*(*t*)*/τ*, in which *f*′(*t*) is the first derivative of *f*(*t*) and *τ*=2 s, as estimated from the decays of the GCAMP6s fluorescent transients^20^. This method efficiently corrects the strong discrepancy between fluorescence and firing time courses due to the slow decay of spike-triggered calcium rises, as we show in simulations based on GCAMP6s kinetic parameters (Supplementary Fig. 3). However, as our simulation also shows, it does not correct for the relatively slow rise time of GCAMP6s, producing a time delay on the order of 70 ms between peak firing rate and peak deconvolved signal (Supplementary Fig. 3). Note that deconvolution is a linear operation and thus cannot be the cause of asymmetric integrals observed for up and down-ramps (Supplementary Note 1). Weak nonlinearities have been observed for the conversion of action potentials into the GCAMP6s signals, most particularly superadditivity of calcium transient amplitudes. As we show that a linear model followed by a nonlinear function cannot explain the observed data (Fig. 6), we can rule out the involvement of GCAMP6s superaddivity in the asymmetry observed for up- and down-ramps. However, in the absence of extensive characterization of GCAMP6s in our imaging conditions, we cannot fully exclude that another uncharacterized nonlinearity of GCAMP6s participates in the asymmetry. The integrals of population and single cluster responses were computed between the time of sound onset and the time of sound offset+500 ms. The normalized difference of integrals (*I*_up_ and *I*_down_) between up- and down-ramps was computed as 2 (*I*_up_−*I*_down_)/(*I*_up_+*I*_down_).

### Analysis of population pattern similarity

Population activity at time *t* of the *i*th repetition of sound *j* was represented by a 4,088-dimension vector 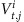 containing the firing rates of all imaged neurons. The similarity between population responses to sound *j* at time *t* and to sound *j′* at time *t′* was computed as the mean correlation between all single-trial vectors pairs *s*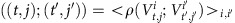, where *ρ* is the Pearson correlation coefficient between two vectors. For (*t,j*)=(*t′,j′*), this measure evaluates the reproducibility of the response patterns across trials (in this case, the pairs *i*=*i′* are excluded from the averaging). In the plotted similarity matrices, similarity was evaluated frame by frame after smoothing the signals with a Gaussian filter (*σ*=60 ms). Note that because trial to trial variability of single neuron responses is very large in mouse auditory cortex^22^, the correlation between single-trial population vectors is generally low (∼0.2). Thus, the average cross-trial correlation (that is, our similarity measure) takes generally low values. However, the idea of this framework is to compare the similarity measure to the self-reproducibility measure. In particular, two population patterns can be considered indistinguishable, given the observed single-trial variability, when their similarity is as high as the individual reproducibility measures (diagonal of the similarity matrix).

### Single-cell clustering analysis

Clustering was used to organize the imaged neuronal responses. Due to the large variability observed in many neurons, this analysis is not exhaustive, but rather aims at identifying principal classes of responses within our data set. Clustering was performed across the 13 imaging sessions, in which we played both white noise and 8 kHz harmonic sounds (3,657 neurons). Response traces for all sounds were smoothed using a Gaussian filter (*σ*=31 ms). Before clustering, we selected significantly responsive (assessed by testing for a difference of the pooled responses to all stimuli against their baseline using a paired Wilcoxon signed-rank test, *P*<0.05) and selective neurons (significant modulation by one of the stimuli, Kruskal–Wallis test, *P*<0.05) neurons. For the 2,343 neurons that passed both tests, the SNR was calculated as 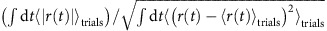. We observed that the SNR distribution was long tailed with a small fraction of cells responding with high SNRs. To base the clustering on the clearest signals, we first selected the 30% of the cells with largest SNR. Using the Euclidean distance on *z* scored response traces (that is, normalized by their standard deviation), as a similarity metric across cells and the ‘furthest distance' as a measure of distance between clusters, we established a hierarchical clustering tree. The tree was thresholded to yield 50 different clusters. This method yielded a large number of small clusters, which after visual inspection appeared to contain noisy responses (hence very dissimilar to other clusters). We therefore excluded clusters containing <10 cells. Applying this criterion, we obtained 13 clusters. Non-clustered cells were then assigned to 1 of the 13 clusters with which they had the highest correlation (Pearson correlation coefficient) provided that this correlation was >0.1. After this procedure, 1,341 neurons were assigned to a cluster while 1,002 cells were not assigned. Inspection of their responses showed that the latter were weakly or non-responsive cells.

### Behaviour

We measured sound salience indirectly by measuring learning speed during associative conditioning. In the first task, water-deprived mice (33 μl g^−1^ per day) were head-fixed and held in a plastic tube on aluminium foil. A 2-s white noise ramp sound (range 60–85 dB) was presented every 6–16 s (uniform distribution) followed by a 1 s test period during which the mouse had to produce at least one lick on a stainless steel water spout to receive a 5 μl water drop. Licks were detected by changes in resistance between the aluminium foil and the water spout. By increasing random lick rates, mice received almost all available rewards within 2–3 days, but the time-locking of licks to the sound increased more slowly (Fig. 7c). Learning speed through which we estimate relative salience of up- and down-ramps was calculated by quantifying the number of post-stimulus licks divided by the number of pre-stimulus licks. The second task was a stimulus avoidance (or Go-NoGo) task. Mice were freely moving in a transparent box equipped with a water spout flanked by an infrared detector. When a mouse approaching the spout was signalled by the detector, a 2-s white noise ramp was played (range 60–85 dB). During the first 4 days, only a rewarded S+ ramp was presented (either up or down, depending on the training group). Mice had to signal their licking by breaking the infrared beam for >1.125 s (licking threshold) during a 1.5 s time window after sound offset to get a reward. All mice reached >80% correct performance on this task after 4 days of training. Then, a distractor S− ramp was introduced with direction opposite to the S+. If mice licked above the S+ licking threshold after the S− was presented, a time out of 8 s was issued in addition to the 5 s interval before the next trial. No time out was issued after incorrect S+ trials. Beam breaks were measured as a continuous voltage signal (Fig. 7e) that was thresholded to compute lick duration. Salience of the sounds in this task could be compared through the time necessary for going from 20 to 80% of plateau performance. Behavioural analyses were all automated thus no animal randomization or experimenter blinding was used.

### Cortical response models

We tested different models to account for the mean cortical response *r*(*t*) to the envelope *s*(*t*) of the intensity-modulated sounds converted to dB SPL (nonlinear function). The linear model (Fig. 2a) corresponds to the convolution of *s*(*t*) with a causal kernel *h(t)*, defined over *t*∈[0; 1] s, that was fitted to the data using the Moore–Penrose pseudo-inverse method^29^. For the adaptation model^31^ (Fig. 2b), an adapted version of the input *s*_*d*_(*t*) was computed as *s*_*d*_(*t*)*=s*(*t*)(*1−d*(*t*)) with *d*(*t*) solution of the differential equation:


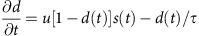


Then a linear kernel was applied to *s*_*d*_(*t*) to fit the cortical response as for the linear model. The best fit of the model to the 2 s white noise ramps was obtained by a brute force search for parameters *u* and *τ*. This fitting approach illustrates our analytical demonstration that both the linear and adaptation model cannot account for asymmetric responses to the up- and down-ramps described in Supplementary Note 1. Note also that the linear temporal filter used here is fully equivalent to a spectro-temporal filter when applied to sounds that have identical spectral content (Supplementary Note 1).

We also tested different models to account for the 13 response clusters *r*_*i*_(*t*) observed in our data set (Fig. 6). For all models, the input was split into ‘loud' *s*_L_(*t*) and ‘quiet' *s*_Q_(*t*) channels computed as sigmoid functions of the input:


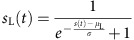



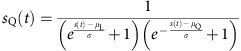


In all models, the three parameters of these two functions were optimized using a brute force approach to best approximate the fit between the final output of the model and the data.

For the LN model, we modelled the response of cluster *i* as


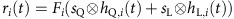


in which *h*_Q*,i*_ and *h*_L*,i*_ are two kernels defined on *t*∈[0; 2] s and *F*_*i*_(*x*)=*a*_*i*_(*x*−*x*_0,*i*_)+*c*_*i*_ if *x*≤*x*_0,*i*_ and *F*_*i*_(*x*)=*b*_*i*_(*x*−*x*_0,*i*_)+*c*_*i*_ if *x*>*x*_0,*i*_ is a monotonous piecewise-linear function. Fitting was done by first determining the kernels that best fit the data before applying the nonlinearity, and then finding the parameters of *F*_*i*_(*x*) that minimize the discrepancy between the sum of the kernel outputs and the data.

For the full nonlinear feature model, a layer of six nonlinear feature detectors (three ‘loud', three ‘quiet') were constructed as 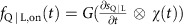, 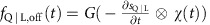 and 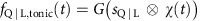
in which 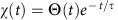, Θ(*t*) is the Heaviside step function, *τ*=0.05 s and *G* is piecewise-linear function (*G*(*x*)=*x*−*θ* if *x*>*θ* and *G*(*x*)=0 otherwise (note that a single threshold value *θ* is used for the six feature detectors). We then modelled the cortical responses as a weighted sum of the two ‘tonic' features (no-kernel) and of the four transient features convolved with linear kernels:


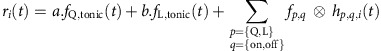


To fit the model, the scalars *a* and *b*, as well as the kernels *h*_*p,q,i*_(*t*) were obtained using linear regression solved exactly (Moore–Penrose pseudo-inverse method) once the nonlinear features had been generated. We either set the threshold *θ* of *G* to zero or optimized it together with the parameters of the ‘loud' and ‘quiet' input channels. For evaluation of the fraction of variance accounted by the model, we first trained the model on a subset of the white noise stimuli (0.25 s constant sounds at 50, 60, 70 and 85 dB+all up- and down-ramps between 50 and 85 dB) and measured the unexplained variance on the response of the model to a test set of white noise stimuli (0.25 s constant sounds at 55, 65 and 80 dB+all up- and down-ramps between 60 and 85 dB).

### Data availability

The data that support the findings of this study and the analysis code are available from the corresponding author upon request.

## Additional information

**How to cite this article:** Deneux, T. *et al*. Temporal asymmetries in auditory coding and perception reflect multi-layered nonlinearities. *Nat. Commun.* 7:12682 doi: 10.1038/ncomms12682 (2016).

## Supplementary Material

Supplementary Information Supplementary Figures 1-5 and Supplementary Note 1
