# Supplementary material for: Temporal asymmetries in auditory coding and perception reflect multi-layered nonlinearities
Source: Nat Commun. 2016 Sep 1;7:12682. doi: 10.1038/ncomms12682 (PMC5025791; doi:10.1038/ncomms12682)
Supplement: Supplementary Information — Supplementary Figures 1-5 and Supplementary Note 1 [file ncomms12682-s1.pdf]

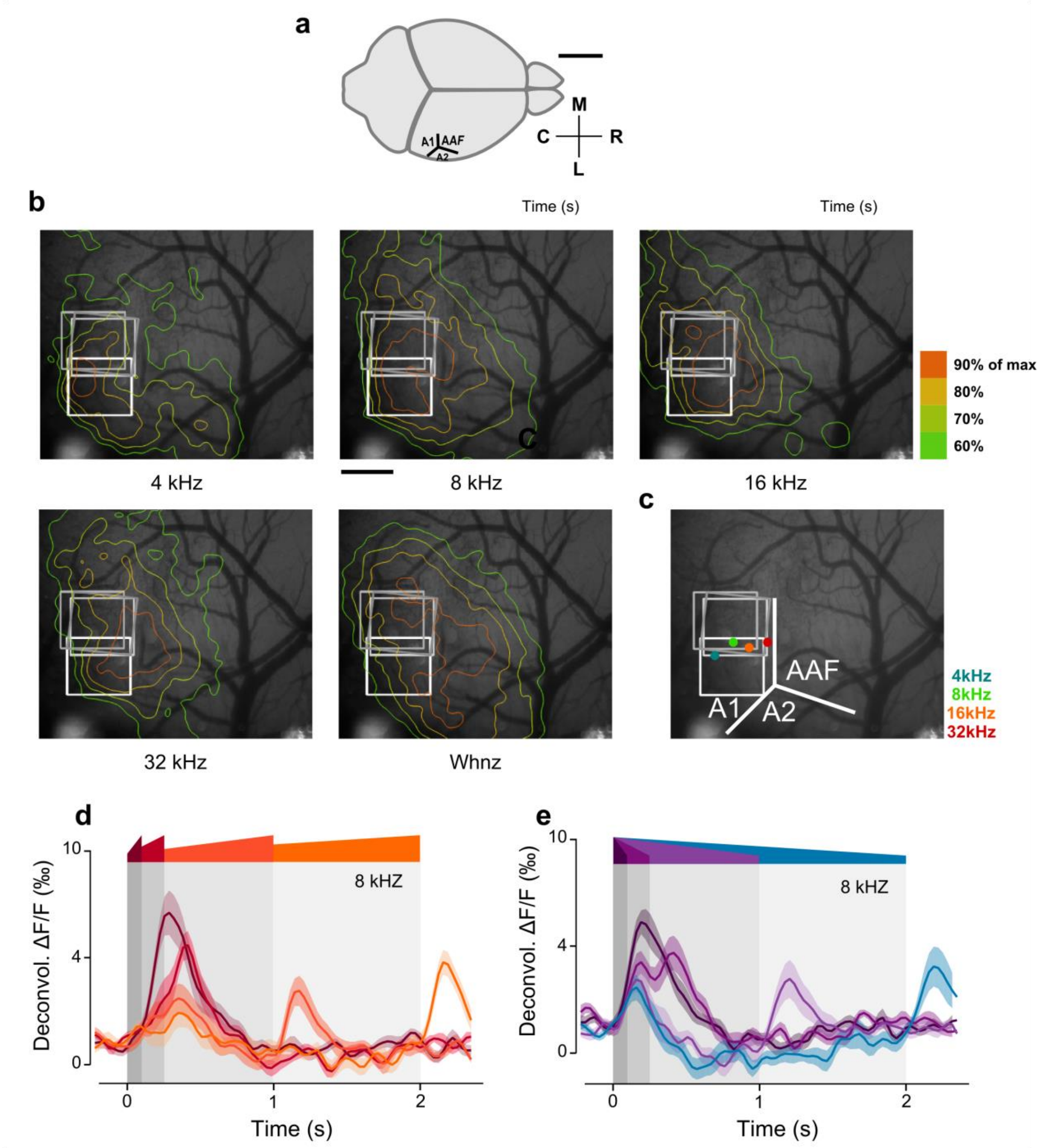

Supplementary Figure 1: Functional localization of the right auditory cortex with intrinsic optical imaging.

a. Localization of the right auditory cortex relative to the mouse brain in mouse 3. Auditory core fields (composed of A1 and AAF) are located on the rostro-caudal axis while secondary fields of the belt region (e.g. A2) are more ventral or dorsal. Scale bar: 5 mm

b. Identification of the auditory cortex and its subfields through intrinsic optical imaging of responses to pure tones. The contour maps superimposed to the blood vessels image represent the ratio of intrinsic signal before and during a 2 s auditory stimulation, here expressed as the percentage of the maximum response for each stimulus. For the animal shown in this example, four two-photon imaging sessions were performed at different location. They are represented as grey rectangles (overlapping locations indicate recordings at different depths) and cover a large part the A1 subfield

coarsely identified from the tonotopic gradient observed in the intrinsic responses (see also **c**). Scale bar: 500 µm

c. A tonotopic gradient perpendicular to the media-lateral axe can be deduced from intrinsic imaging signals. Each dot represents the centroid of the area in which the intrinsic signal is within 90% of the maximum response for each sound frequency. This rostro-caudal gradient from low-frequency (blue) to high frequency tuning (red) correspond to the A1 subfield[21]. The mirror symmetric gradient from AAF can be deduced from the anterior local response peak seen in the response to 4 kHz. These gradients were used to coarsely identify the location of auditory cortex subfields.

d. Mean deconvolved calcium signals (i.e. estimated firing rate) for 8kHz up-ramps of duration 100ms, 250ms, 1s and 2s (range 60-85 dB SPL, shading indicates SEM across imaging sessions, n=13).

e. Same as **d**. for 8kHz down-ramps of duration 100ms, 250ms, 1s and 2s.

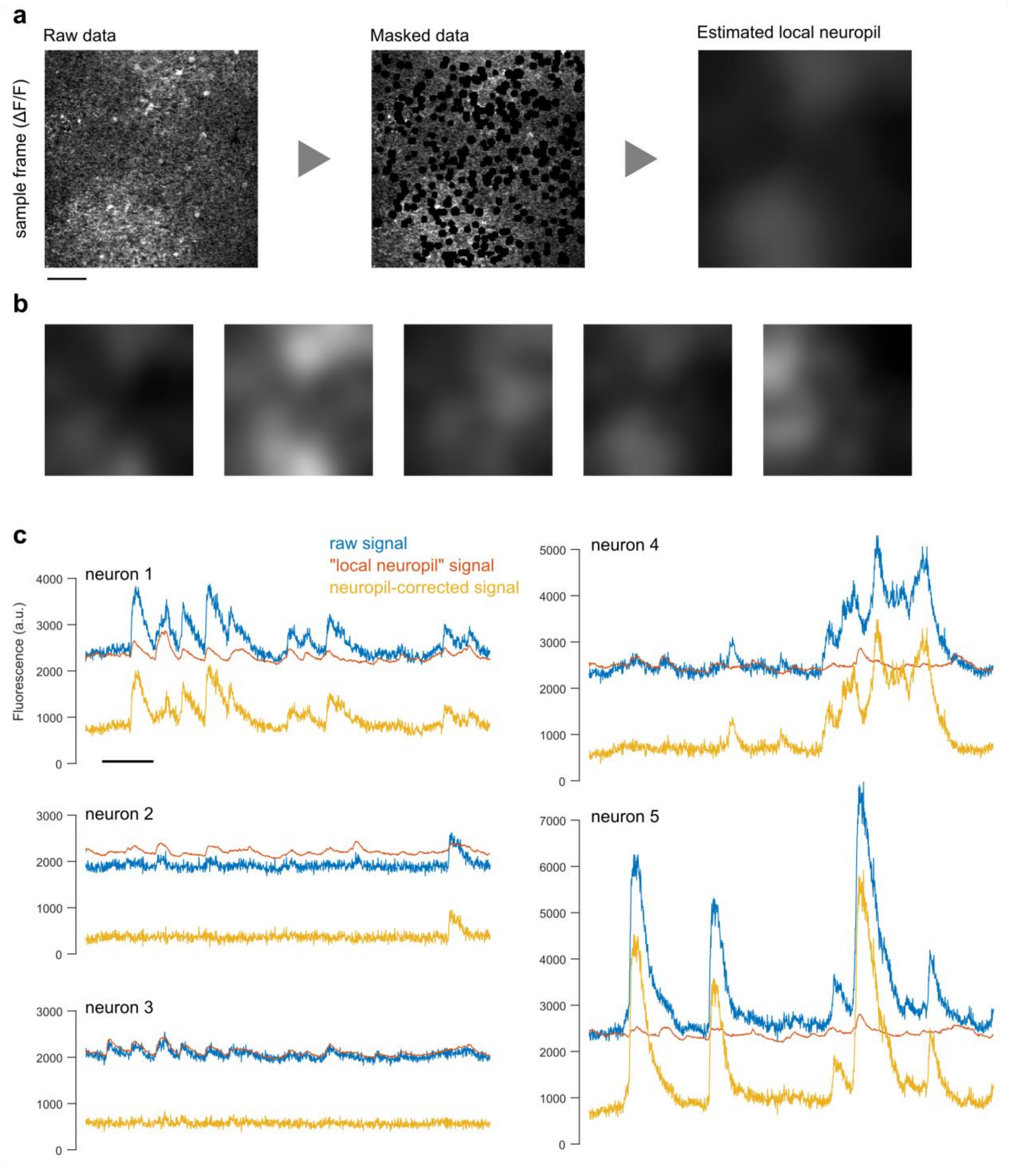

Supplementary Figure 2: Correction for neuropil contamination.

a. Neuropil estimation method: each individual frame is multiplied with a mask that avoids all selected neurons, and appropriate smoothing (see Methods) is used to fill-in image parts that were masked out. This permits to estimate the average neuropil signal at the location of the neuron. Scale bar: 100 µm

b. Sample images of local neuropil estimation from the same imaging session showing clear variations across different time points and spatial locations.

c. Raw signals from five individual neurons (in blue) and corresponding local neuropil signals (in red) extracted from the same ROIs. The neuropil-corrected signals (yellow) are obtained by subtracting from the raw signal a scaled version (by 0.7) of the local neuropil signals. For some neurons (e.g. bottom left) all the signal present in the raw data is removed, while for others simultaneously imaged neurons (e.g. bottom right) the signals are little affected by the correction. Scale bar 5 s.

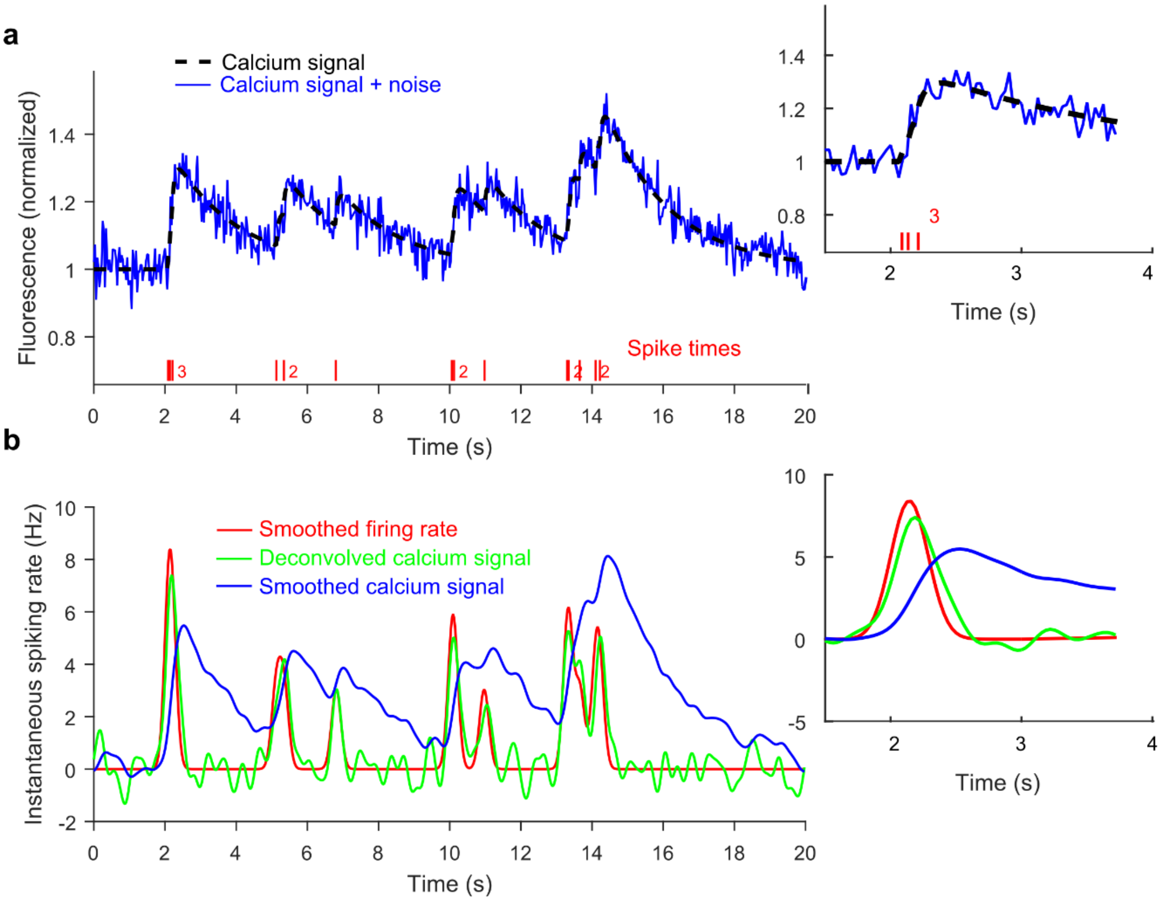

Supplementary Figure 3: Deconvolution of calcium signals: simulations.

a. Simulated GCAMP6s fluorescence (black line) resulting from the train of spike shown below (red bars). The GCAMP6s signal resulting from a single spike is here modeled as double exponential with a unitary calcium increase a of 11.3%, a rise time τ_{on} of 70 ms and an exponential decay τ of 1.87s as described in mouse visual cortex[1] (specifically, $F(t)/F_0 = \sum_{t_{spike}<t} a\left(1-\exp\left(1-\frac{t-t_{spike}}{\tau_{on}}\right)\right)\exp\left(\frac{t-t_{spike}}{\tau}\right)$). The blue line corresponds to the simulated signal superposed with white noise. Magnified signal in the inset highlights the temporal delay of the fluorescence peak compared to spikes due to the 70ms rise time.

b. Applying our linear deconvolution algorithm (see Methods) followed with Gaussian smoothing to the noisy fluorescence signal shown in **a.** yields an estimate of the time course of the instantaneous firing rate (green) which matches the smoothed instantaneous rate (red) much better than smoothed calcium signal (blue, the scale is hand-adjusted to match the rate signals). Correlation of the smoothed firing rate is much higher with the deconvolved calcium signals (0.91) than with the smoothed raw calcium signal (0.21), despite the fact that the deconvolution ignored the slow rise time of GCAMP6s, which results in a slight delay of the rate estimate, as can be seen in the inset. Note that this simulation and estimation proves robustness of the deconvolution to the mismatch between the assumed and actual models of calcium signals in terms of raise time (which is inexistent in the deconvolution), but also in terms of decay time, as the decay time used in the stimulation is of 1.87s, whereas it is assumed by the deconvolution to be of 2s (see Methods).

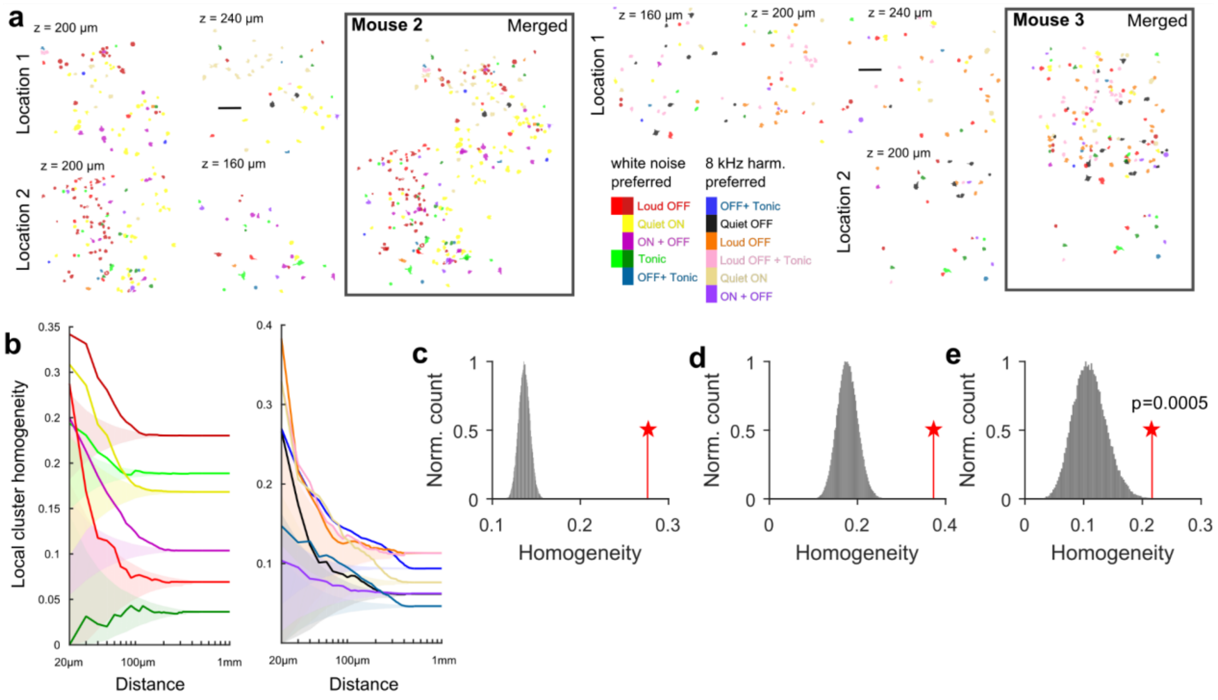

Supplementary Figure 4: Functional cell assembly organization.

a. Localizations of the cells belonging to the different identified clusters in four imaging sessions performed at two different horizontal localizations and different depths (z) across several days in mice 2 and 3. The localizations of mouse 3 recordings within the auditory cortex can be found in **Supplementary Fig. 1**. The color-code used for the different clusters is consistent with **Fig. 4** and **Fig. 5** (see colorbar). On the right, the localization of all cells is shown in a horizontally-mapped z-projection. Scale bars: 100 µm.

b. Single cluster homogeneity for the 13 identified clusters (see color code in **a**) when the radius of analysis is varied. The shaded areas represent the range of values for the homogeneity index observed in 99% of the cell identity shufflings (bootstrap). Homogeneity is maximal at small distances and drop below statistical dependency at about 100µm distance (depending on clusters); note that in the shuffled data the average homogeneity is the same at all distances by construction, however the variability is higher at small distances because the number of neuron pairs at small distances is much smaller than for large distances.

c-e. Spatial clustering is present across different mice and recordings. Distributions of a global homogeneity index (mean probability for the neighbors of any given neuron within an 30 µm radius to belong to the same functional cluster, averaged over neurons from all clusters and within single mice) for 100,000 shufflings of the cell identities (bootstrap) compared to the experimental values for different mice.

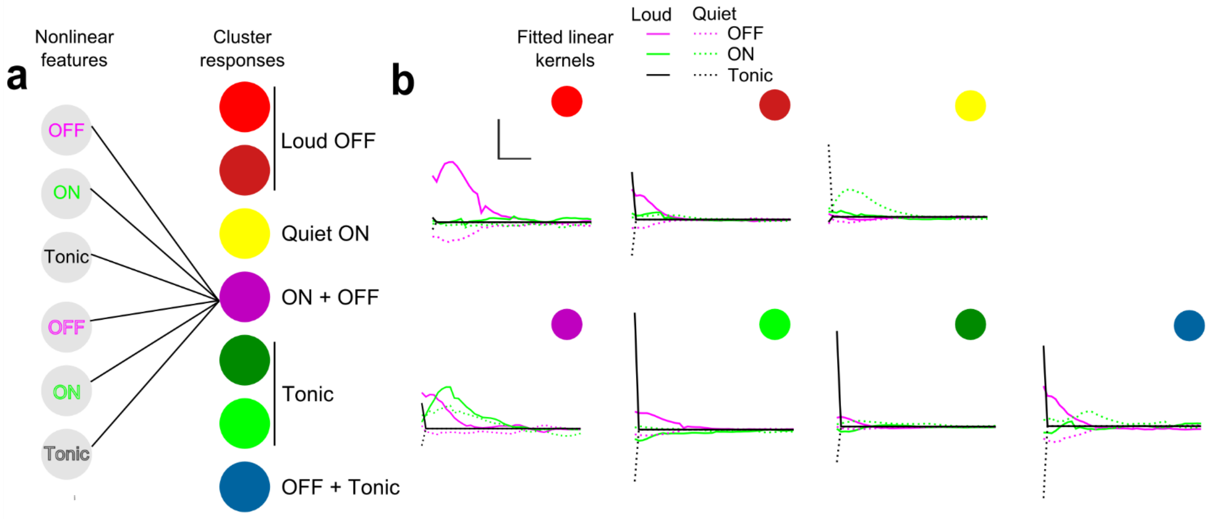

Supplementary Figure 5: Fitted kernels for the multilayer feature model

a. Schematics of the last two layers of the multilayer model.

b. Fitted kernels linking the input feature layer to the seven clusters showing preference for white noise. Green (resp. magenta) traces represent kernels from ON (resp. OFF) input features (plain line: loud; dashed line: quiet). Black peaks represent the weight of the constant input (Loud Tonic, continuous line, Quiet Tonic). These plots clearly show that certain clusters (e.g. red) mostly reflect a single input feature, while others are better modeled by mixed inputs. Moreover the time-course of the kernels reflect simple regular transient functions with temporal phasic temporal profile with a decay time constant of 200 to 300 ms resembling which are compatible with biological slow and polysynaptic post-synaptic potentials e.g. coming from au upstream neuronal population. This suggests that the model effectively summarizes the summation of different functional inputs (however complex the real presynaptic connectivity might be) in the cortical neurons and does not perform extensive overfitting. Horizontal scale bar: 0.25s. Vertical scale bar: 0.1 arbitrary units.

Supplementary Note 1

1 General question: transformations which preserve of the integral of their output after time-reversal of their input

For any input signal $s(t)$, defined as an integrable function on $\mathbb{R}$, we are interested in transformations F from the space of integrable function to itself, for which the time integral of the output signal is invariant with respect to time-reversal, i.e. the transformation that satisfy the property P_0:

$$\int_{-\infty}^{+\infty} F\left[s(t)\right] dt = \int_{-\infty}^{+\infty} F\left[s(-t)\right] dt$$

We here describe analytical proofs of this property for specific transformations or classes of transformation. Note that in the following, the notation $\int$ is used for $\int_{-\infty}^{+\infty}$.

2 Effect of an arbitrary function applied to the input before the transformation

It is interesting to mention, that if F is a transformation that satisfy P_0, this applies to any integrable function on $\mathbb{R}$. So for any function $f : x \to f(x)$ from $\mathbb{R}$ to $\mathbb{R}$ such that $f(s(t))$ is still integrable, the transformation $F[f(s(t))]$ also satisfies P_0. In other words, any function (including non-linear functions) applied to the input signal before the transformation does not affect the invariance of the output integrals to a time reversal.

3 Invariance for a linear transformation

A general linear transformation of a function $s(t)$, invariant by translation (i.e. the transformation does not depend on the absolute time at which is occurs) can be written as a convolution with a filter $h(t)$.

$$F : s(t) \to \int h(t-u)s(u)du$$

For such a transformation the integral of the time-reversed signal is:

$$\int F[s(-t)] = \int\int h(t-u)s(-u)dtdu = \int s(-u)du \int h(t-u)dt$$

So by setting $t' = t - u$ and then $u' = -u$ one easily obtains the equality of the integrals:

$$\int F[s(-t)] = \left(\int s(u')du'\right)\left(\int h(t')dt'\right) = \int F[s(t)]$$

4 Case of STRF filters

In the particular case of a STRF filter, the input signal is the spectrogram $\hat{s}(t, f)$ of the signal $s(t)$. The response $r(t)$ of a neuron predicted by its associated spectro-temporal receptive field, is computed by first convolving the spectro-temporal kernel $STRF(t, f)$ with $\hat{s}(t, f)$

$$\hat{r}(t, f) = \int duSTRF(u, f)\hat{s}(t-u, f)$$

This transformation is linear and invariant by time translation, thus for all frequencies f the time integral of $\hat{r}(t,f)$ is not affected by time-reversal. $r(t) = \int \hat{r}(t,f)df$ corresponds to the sum of $\hat{r}(t,f)$ over all frequencies f. This integration step is independant of time and thus is also unaffected by time-reversal. Therefore the integral of STRF predictions of a neuron's response is in all cases unaffected by time reversal of the stimulus.

In addition in the particular case of the stimuli used in this study which have a frequency content that is invariant over time, the spectrogramm can be written as a product of a spectral and enveloppe component $\hat{s}(t,f) = g(f)S(t)$. In this case:

$$r(t) = \int duS(t-u) \int g(f)STRF(u,f)df = \int duS(t-u)\tilde{STRF}(u)$$

Thus the STRF framework simplifies for this particular case to convolution with a frequency independent effective kernel $\tilde{STRF}(t)$ valid for a particular frequency content. This justifies that the use of a frequency independant kernel to fit, within the STRF framework, the neuronal responses to enveloppe variations of white noise stimuli.

5 Invariance for the synaptic depression model

The model of synaptic depression is defined by David et al. (2009) as a discrete time equation for a depression variable d:

$$d(t+1) = d(t) + s(t)[1-d(t)]u - d(t)/\tau$$

from which the output signal is obtained as:

$$s_d(t) = s(t)(1-d(t))$$

The first equation yields in continuous time:

$$d'(t) + [1/\tau + us(t)]d(t) = us(t)$$

in which $d'(t)$ is the first derivative of $d(t)$. If we take that $s(t) = 0$ for $t \leq 0$, (i.e. the signal starts at $t = 0$) the solution of this first order linear equation can be written as:

$$d(t) = u \int s(x)e^{-\int_x^t (1/\tau + us(v))dv}\theta(t-x)dx$$

in which θ is the Heaviside step function.

Because $s_d(t) = s(t) - s(t)d(t)$ the invariance to time-reversal will be obtained if and only if $A_s = \int s(t)d(t)$ is invariant to time-reversal. For the forward signal A_s (normalized by u) writes as:

$$A_{s+} = \iint dxdts(t)s(x)e^{-\int_x^t (1/\tau + us(v))dv}\theta(t-x)$$

And for the time-reversed signal it writes as:

$$A_{s-} = \iint dtdxs(-t)s(-x)e^{-\int_x^t (1/\tau + us(-v))dv}\theta(t-x)$$

Setting $t' = -t$, $v' = -v$ and $x' = -x$ yields,

$$A_{s-} = \iint dt'dx's(t')s(x')e^{-\int_{t'}^{x'} (1/\tau + us(v'))dv'}\theta(x'-t')$$

In the expression above, the x' and t' are equivalent. Hence:

$$A_{s-} = \iint dxdts(x)s(t)e^{-\int_x^t (1/\tau + us(v))dv}\theta(t-x) = A_{s+}$$

proving that the output integral of the synaptic depression model is invariant to time-reversal of the input signal despite its nonlinearity.

6 Some sufficient conditions for a linear non-linear transformation (LN model)

We now suppose that the transformation F is a linear filter of kernel h followed by a non-linear function f, i.e.

$$F : s(t) \to f\left(\int s(t-u)h(u)du\right)$$

In this case two sufficient conditions for P_0 can be derived.

Sufficient condition 1 If h has a vertical symmetry (i.e. it exists x_0 such that for all x, $h(x - x_0) = h(x_0 - x)$) then F satisfies P_0.

Proof Three changes of variable: $u \to x_0 - v$ followed by $v - x0 \to u'$ and $t \to t'$ yield the equality.

$$\int f\left(\int s(-t+u)h(u)du\right)dt = \int f\left(\int s(-t+x_0-v)h(x_0-v)dv\right)dt = \int f\left(\int s(t'-u')h(u')du'\right)dt'$$

Sufficient condition 2 If h has a central symmetry (i.e. it exists x_0 such that for all x, $h(x - x_0) = -h(x_0 - x)$) and if $f(x) = 0$ for $x \le 0$ and $f(x) = x$ for $x > 0$, then F satisfies P_0.

Proof If h has central symmetry then $\int h(u)du = 0$ and $\int\int s(u)h(t + u)dudt = 0$. So if we call $\mathbb{H}^+$ the sub-ensemble of $\mathbb{R}$ in which $\int s(u)h(t + u)du > 0$ and $\mathbb{H}^-$ its complementary in $\mathbb{R}$, we have $\int_{\mathbb{H}^+}\int s(u)h(t+u)dudt = -\int_{\mathbb{H}^-}\int s(u)h(t+u)dudt$. The proof then comes from the fact that the integral of the time-reversed signal can be re-written as:

$$\int f\left(\int s(-u)h(t-u)du\right)dt = \int f\left(\int s(u')h(t+u')du'\right)dt' = \int_{\mathbb{H}^+}\int s(u')h(t+u')du'dt'$$

an that using the central symmetry of h we get for the integral of the forward signal:

$$\int f\left(\int s(u)h(t-u)du\right)dt = \int f\left(\int s(u)h(-t'-u)du\right)dt' = -\int_{\mathbb{H}^-}\int s(u)h(t'+u)dudt'$$

which thanks to the above mentioned equality leads to the proof.

7 Conclusions

In our experiments, we have observed that ramping-up sounds produce cortical responses with a larger time-integral that ramping-down sounds, although the time-integral of the envelop of the two sounds are the same. The above proofs show that models with a non-linear intensity scaling function followed by a linear filter are mathematically unable to explain this property in the general case. Moreover, the addition of a previously described non-linear adaptation model is also mathematically unable to explain the data.

Lastly, we show that models constructed with a linear filter followed by a non-linearity (LN models) will not be able to reproduce the observed experimental property if the kernel of the filter is has vertical-symmetry or in the case of very simple rectifying non-linearity if the the kernel has a central symmetry.

Note that, in other conditions, LN-models actually can produce unequal output time-integrals although input integrals are equal. Nevertheless, LN-models are unable to reproduce the temporal profile of recorded neuronal responses (see Fig. 7), because these responses encode features that are incompatible in a LN-model. For example, a linear filter cannot respond positively both at the onset and at the offset of a positive signal (as many neurons in auditory cortex do, e.g. ON-OFF cluster), because linear on-response (resp. off-response) filters also respond negatively at an offset (resp. onset). Hence the addition of linear on- and off-response filters produces overall no output which no subsequent nonlinear function can compensate. To model neurons that respond positively both to onsets and offsets, it is necessary to insert a nonlinearity before summing the two features as we did it our multilayer non-linear features model (Fig. 7).
